# Physical activity level in people with age related white matter changes correlates to better motor performance, lower comorbidity and higher cognitive level

**DOI:** 10.1186/s12877-017-0535-z

**Published:** 2017-07-12

**Authors:** Anna F. Pettersson, Lars-Olof Wahlund, Lena Bronge, Elisabeth Olsson, Kaarina Amberla, Hansjoerg Baezner, Milita Crisby

**Affiliations:** 1 Division of Physiotherapy, Karolinska Institutet, Neurobiology Care Sciences and Society, 23 100, 141 83 Huddinge, Sweden; 2 Division of Clinical Geriatrics, Karolinska Institutet, Neurobiology Care Sciences and Society, Huddinge, Sweden; 3 Division of Radiology, CLINTEC Department, Karolinska Institutet, Huddinge, Sweden; 4 Department of Clinical Psychology, Helsinki University, Helsinki, Finland; 5 Department of Neurology, Universitätsklinikum Mannheim, University of Heidelberg, Heidelberg, Germany

**Keywords:** Cognition, Gait, Health, Motor function, Physical activity

## Abstract

**Background:**

Physical activity plays a pivotal role in the development of disability and may modify the negative effect of vascular risk factors on progression of both cardio and cerebrovascular disorders. The aim of this study was to evaluate the activity level in people with age-related white matter changes as identified on magnetic resonance imaging (MRI) in relation to motor performance, cognition and perceived health.

**Methods:**

Data came from the first year follow up of one participating centers of the LADIS study. Fifty one subjects were first enrolled in the study. Complete first year follow up data was available for 41 subjects. Information on comorbidity, physical activity level, physical function, cognition, level of white matter changes and perceived health was collected. Physical activity level was classified with a yes or no question and with the Frenchay Activities Index (FAI).

**Results:**

Only 36% of the subjects in this study were physically active according to the yes/no question. 27.5% of the subjects were active according to the FAI score which evaluates the everyday activities. Being active discriminated subjects with better physical function. Subjects active according to the FAI score had a higher cognitive level (*p* ≤ 0.01), lower comorbidity (*p* = 0.02) and performed better on all motor function tasks as assessed by walking speed (*p* ≤ 0.01) and the Short Physical Performance battery (SPPB) (*p* ≤ 0.01).

**Conclusions:**

Being physically active seems to be a long term protective factor. In our study, the majority of subjects with Age Related White Mattter Changes (ARWMC) with no or mild Instrumental Activity of Daily Living (IADL) disability did not attain recommended level of activity at first year follow up. Whether or not increasing physical activity may slow down cognitive decline and lessen development of disability in physically inactive subjects with manifest ARWC remains to be studied. Trial registration: not applicable.

## Background

Cognitive decline is among the most feared and economically costly sequela of ageing [1, 2]. Cognitive decline is associated with a reduction in the ability to perform everyday tasks, make important decisions, and thus live independently [3, 4]. Ageing is a complex process and appears to affect white matter integrity and functional connectivity in the brain [5]. Physical fitness and possession of the APOE e4 allele have been shown to be incrementally associated with cognitive change at statistically significant level [6].

Higher levels of physical activity have been associated with larger gray-matter volumes [7]. Furthermore, cardiorespiratory fitness affects white-matter integrity [8, 9], which may consequently have positive effects on functional brain activity in old age [10].

Moderate physical activity for at least 30 min, no less than 3 times/week has been reported to maintain cardiovascular fitness [11]. This level of exercise has shown to decrease vascular risk factors and the incidence of coronary heart disease [12]. In addition, low intensity physical activity renders other health benefits such as maintaining physical function, a higher quality of life, and wellbeing [13]. Even low intensity physical activity has been shown to be associated with larger hippocampus volume in older adults [14]. Many of our daily activities include activities that are dependent on lower extremity function, which has been found to be able to predict short-term mortality and nursing home admission in older adults [15–17]. Gait speed alone has been found to predict incident disability in previously non-disabled older people [18].

Age related brain white matter changes, (ARWMC), are hyperintense lesions observed on T2-weighted images on brain magnetic resonance imaging (MRI). ARWMC are common findings in older people and have been associated with cognitive impairment as well as gait disturbances [19, 20]. Cerebral ischemia related to deep cerebral small vessels of the brain due to aging, arterial hypertension and other vascular risk factors has been suggested as possible causes of ARWMC [21, 22]. The severity of ARWMC on brain MRI has been described by Fazekas et al. as; grade I (punctuate changes), grade II (early confluent changes) and grade III (severe confluent changes) [23]. Gait disturbances may lead to limitations in activities and a restricted ability to participate in life situations and subsequently lead to an inactive life style and increased risk of falls [19, 24, 25]. This has important implications since inactive older people with low physiologic reserves are more vulnerable to disease events and subsequent disability [26, 27]. The present study evaluates cross sectional data of activity level in people with ARWMC in relation to motor performance, cognition and perceived health.

## Methods

### Design

In this cross-sectional study data came from the first year follow up of one of the participating centres of the Leukoaraiosis and Disability study (LADIS) [28]. In brief the LADIS study is a multicentre and multinational longitudinal study with the aim to examine the net contribution of various degrees of ARWMC on the development of disability in older people.

### Subjects

**Fig. 1:**
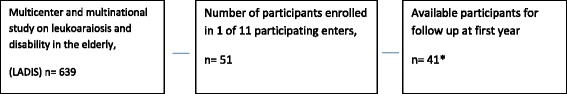
Participant flow chart. *not available for examination n = 4, drop out n = 1, died n = 1, followed up by telephone interview only n = 4

LADIS participants were at entry between 65 and 84 years of age. They had no or mild disability as defined by the Instrumental Activities of Daily Living (IADL). All of them had a contactable informant and had agreed to sign an informed consent prior to participation in the study. Subjects were recruited between 2001 and 2002 through the radiology department where they had presented with cerebral subcortical white matter changes on MRI of any degree. Two of the subjects had been referred to MRI due to cognitive impairment; another five subjects had been referred to MRI due to minor stroke and the remaining subjects with ARWMC were incidentally detected. Subjects were excluded if they were judged likely to drop out due to any severe disease, unable to give informed consent or inability to undergo further MRI scan. Based on the inclusion and exclusion criteria 51 subjects were enrolled in the study. At the first year follow up, data was available for 41 subjects, Fig. 1. The study was approved be the local ethics committee, reference number 01–316.

### Materials and procedure

Subjects underwent a thorough examination according to the LADIS protocol, which has been described in detail elsewhere [28]. In summary these examinations included medical history, social background and assessments of physical and cognitive function and brain MR. The LADIS study includes data on subjects from all European centers up to a 3 year follow-up period [28]. The present study is a sub-study that includes cross- sectional data assessed at the first year follow up at Karolinska University Hospital, Huddinge, Sweden. For the purpose of this study an additional evaluation of everyday activities was performed.

### Medical history/co-morbidity

Information on comorbidity came from interviewing the patient and the informant, and from the medical records. Comorbidities included cardiovascular risk factors and age related comorbidities known as possible determinants of disability in the elderly. All of these were defined according to the currently most widely accepted criteria, selected after a systematic literature search reported in detail elsewhere [28].

### Medical history/physical activity

Physical activity level was classified with a yes or no question. Being physically active was defined according to the American Heart Association (AHA) Scientific definition, (i.e. at least 30 min of activity and at least 3 times/week) [11].

### Additional assessment of every day physical activity

We were especially interested to look at lower intensities of physical activities since this also may have health benefits for older people. Consequently, for the purpose of the present study the activity level was further assessed with the Frenchay Activities Index (FAI) [29], a questionnaire reflecting lower intensity activities including questions about the frequency of domestic chores, leisure/work and outdoor activities. A high score indicates higher level of activity (max 45). The FAI has been validated in a general population aged 16 and over [30].

### Assessment of cognitive function

General cognitive ability was assessed using the Mini Mental State Examination score (MMSE) [31].

### Assessment of physical function

Physical function was assessed using the Short Physical Performance Battery (SPBB) that includes tests of standing balance, walking speed and time to rise from a chair five times. Subjects are scored 0–4 depending on time performance, with 0 representing the inability to complete the task and 4 representing the highest level of performance. Maximum score is 12.

In addition, walking speed (m/s) was recorded by measuring the free walking speed over an 8 m course [32].

### Evaluation of perceived health

The subjects’ perception of own health status was evaluated using the visual analogue rating scale (VAS) from the EuroQoul/EQ5D [33]. The EQ5D VAS is a vertical 20 cm visual analogue scale similar to a thermometer. It ranges from worst imaginable health state, 0, to best imaginable health state, 100.

### MRI scanning/rating of ARWMC

All subjects had been examined by MRI according to a standard protocol. The degree of ARWMC severity was rated by one of the co-authors, an experienced consultant in neuroradiology (LB), using the visual scale of Fazekas as grade 1 (punctuate), grade 2 (early confluent), grade 3 (confluent) [23]. ARWMC of grade one was denoted as mild, grade 2 as moderate and grade 3 as severe [28]. Details on the MRI procedure and rating of ARMC are published elsewhere [29].

Subjects characteristics are shown in Table 1.

**Table 1:** Characteristics, all subjects ( *n* = 41). Median (range), Mean ± SD

Number of subjects	41
Age (yrs)	75 ± 4.9
Sex (Males/Females)	20/21
Mini Mental State Examination, MMSE (score)	27 (20–30)
Number of comorbidities	1.6 ± 1.4
Number of persons active according to AHA definition yes/no	15/26
The Frenchay Activities Index, FAI (score)	26 (6–33)
Short Physical Performance Battery, SPPB (score)	9 (3–12)
Chair- stands (s)	17,4 ± 4.7
Walking speed (mean m/s)	1.01 ± 0.2
Euro-Quol, EQ5D (median VAS score)	75 (30–95)

### Statistical analysis

The subject sample was divided in subjects who were physically active as defined according to the AHA scientific definition [11] and less physically active respectively. In addition, we aimed to study those who were defined active at lower intensities using the FAI score that captures domestic chores, leisure/work and outdoor activities. The maximum score of FAI is 45 which indicate a higher level of such activities. For the purpose of this study we wanted to discriminate between high performers and low performers thus subjects who had a FAI score ≥ the median of the whole group were considered active and those subjects below the median were consequently considered inactive. In the analysis, the FAI Score was dichotomised taking the value zero for values below the median/mean and one for values equal to and above the median/mean. The Mann-Whitney U test was used to look at differences between physically active and physically inactive.

Differences among subjects with various degrees of ARWMC was assessed using Kruskal-Wallis ANOVA.

The variables FAI, SPPB, EQ5D, MMSE, age and number of comorbidities were dichotomised, taking the value zero for values below the median/mean and one for values equal to and above the median/mean. Stepwise logistic and stepwise linear regression analysis were then used to evaluate to what extent physical function (SPPB, walking speed) and health (EQ5D) could be explained by the activity level, degree of ARWMC, age, sex, cognition (MMSE score) and co-morbidity.

## Results

According to the AHA scientific definition 36% (*n* = 15) were physically active while 27.5% (*n* = 11) were active according to the FAI score (every day activities). Subjects being physically active according to the AHA scientific definition had significantly better SPPB-scores, (*p*-value 0.01). Subjects active according to the FAI score had a higher cognitive level (p = ≤ 0.01), less comorbidity (*p* = 0.02) and performed better on all motor function tasks such as the walking speed (*p* ≤ 0.01) and the SPPB score (*p* ≤ 0.01) as shown in Table 2.

**Table 2:** Differences in age, sex, co-morbidity, cognitive and physical function and health in active/inactive subjects according to FAI ( *n* = 40) Median (range), Mean ± SD

Variable	Physically active *n* = 11	Physically less active *n* = 29	*p* -value
Age (yrs)	74 ± 5.4	77 ± 3.5	0.12
Sex (M/F)	9/12	12/7	0.27
Co-morbidities (n)	1.0 ± 1.1	2.2 ± 1.5	0.02
MMSE (score)	29 (21–30)	25 (20–30)	≤ 0.01
SPPB (score)	10 (7–12)	7 (3–11)	≤ 0.01
Walking speed (m/s)	1.1 ± 0.2	0.9 ± 0.2	≤ 0.01
EQ5D (VAS score)	80 (30–95)	70 (30–90)	0.24

### Degree of ARWMC

There were no significant differences regarding age, sex, cognitive level, activity level, motor performance or perceived health between subjects with different degrees of ARWMC.

### Tests of physical function

#### SPPB

Stepwise logistic regression analyses showed that cognitive level as measured with MMSE and the number of co-morbidities was significantly related to the performance on the SPPB score (*p*-value 0.01 and 0.02 respectively).

The OR for having a SPPB score equal to or above the median was 1.4 times higher for those with a higher cognitive level as measured with MMSE, (95% confidence interval, 1.07–1.85).

Subjects with co-morbidities equal to or above the mean for the whole group were less likely to perform better on SPPB (OR 0.48, 95% confidence interval 0.25–0.91).

#### Walking speed

Walking speed was significantly related to the subjects’ cognitive level as assessed by the MMSE (*p* < 0,005). In addition, the walking speed correlated to the number of comorbidities and moderate degree of ARWMC (*p*-value 0.02 and 0.04 respectively).

The MMSE scores, number of co-morbidities and degree of ARWMC appeared to be three independent variables which explained 34% of the variation in the walking speed.

### Perceived health

#### EQ5D

Only co-morbidities were significantly related to perceived health as assessed with the EQ5D (*p* < 0,005). Subjects with a number of co-morbidities equal to or above the mean (m = 1.6 ± 1.4) were less likely to score high on the EQ5D, (OR = 0.39; 95% confidence interval: 0.21–0.73).

## Discussion

The majority of the subjects in this study were not active at the suggested level according to the AHA statement. There was a clear association between the activity level of the subjects according to the AHA’s definition and to better physical function such as motor performance and walking speed. A higher degree of physical activity has been shown to attenuate age-related myelin declines in portions of the corpus callosum that interconnect homologous premotor cortex regions involved in motor planning [8]. A previous 3-year prospective study has shown that both sports and non-sports activities (walking and household) have been found to be a determinant of change in mobility performance. Indeed, this effect was independent of presence of chronic disease. Furthermore, the study showed that the subjects who started being active during follow up had a lesser degree of decline in mobility performance compared to those who remained at the same activity level [34].

We also found that the activity level of the subjects correlated to the degree of ARWMC. The positive correlation seen between physical activity and motor performance, cognition and walking speed remained significant even after correction for the demographic variables and were independent of the reported gait disturbances and previous falls. Studies have shown a clear association between white matter integrity and functional connectivity in the brain [35] and being physically active appears to be protective for maintaining white matter integrity [36]. These protective effects could be partly due to the positive effects seen in the regulation of blood pressure and improvements of the glucose metabolism [37, 38].

A major limitation of this study is its’ small sample size. The cross sectional design also makes it difficult to determine the temporal relationship between exposure and outcome and does not allow to draw conclusions on causality.However, it is reasonable to consider the possibility that comorbidity, physical activity, physical function and cognition are interrelated. Co-morbidities may incorporate factors that are perceived as barriers for taking part in physical activity [39]. Such factors may be tiredness, feelings of discomfort or pain. This may lead to a downward spiral of decreasing activity level eventually causing motor disability. Moreover, many of the co-morbidities were cardiovascular risk factors that are thought to play a role in the development of ischemic events and subsequent ARWMC and impaired cognitive function [21, 22]. Executive functions are often impaired in subjects with ARWMC, decrements in executive function may cause less initiative to physical activity [22].

Physical activity has previously been linked to physical function [40, 41] and cognition [42]. In a recent publication from the whole cohort included in the LADIS study, being physically active was associated with reduced risk of cognitive impairment and reduced in more than half, the risk for vascular dementia. However, physical activity did not influence evolution of Alzheimer dementia [43].

## Conclusions

Being physically active seems to be a long term protective factor for cognitive function. In our study, the majority of subjects with ARWMC with no or mild IADL disability did not attain recommended level of activity at first year follow up. Whether or not increasing physical activity may slow down cognitive decline and lessen development of disability in physically inactive subjects with manifest ARWC remains to be studied.

## Abbreviations

AHA: American Heart Association
ARWMC: Age Related White matters Changes
FAI: Frenchay Activities Index
IADL: Instrumental Activities of Daily Living
LADIS: Leukoaraiosis and Disability study
MMSE: Mini Mental State Examination score
MRI: Magnetic Resonance Imaging
SPBB: Short Physical Performance Battery
VAS: Visual Analogue rating Scale
